# Pulmonary Rehabilitation Program Is an Effective Treatment Approach for Post-COVID-19 Syndrome Patients

**DOI:** 10.3390/jcm13185542

**Published:** 2024-09-19

**Authors:** Karina Pádua, Karissa Yasmim Araújo Rosa, Silvania Leal, Iransé Oliveira Silva, Rodrigo Franco de Oliveira, Deise Aparecida de Almeida Pires Oliveira, Luís Vicente Oliveira, Dante Brasil Santos

**Affiliations:** 1 Program in Human Movement and Rehabilitation of the Anápolis University Center, Main Campus Unit, University Av km 3.5, Bloco B2, sl 501, Anápolis 75083-515, GO, Brazil; 2 Biosciences and Human Movement Laboratory, UNIRIO, Institute of Biosciences, Xavier Sigaud St., 290 2nd Floor, Urca, Rio de Janeiro 22290-180, RJ, Brazil; 3 Reference Center for Neuromuscular Diseases, Hospital de Apoio de Brasília, Brasília 70684-831, DF, Brazil; 4 Pulmonary Rehabilitation Program, Hospital Universitário de Brasília—Av L2norte SGAN 604/605, Universidade de Brasília, Brasília 70840-901, DF, Brazil

**Keywords:** COVID-19, pulmonary rehabilitation program, functional status, 6-min walk test, muscular strength

## Abstract

**Background/Objectives:** Patients with post-COVID-2019 syndrome may have reduced functional capacity and physical activity levels. The pulmonary rehabilitation program (PRP)—an exercise training program—is designed to restore these functions and has been shown to improve dyspnea, exercise capacity, and other measures in these patients. This study aimed to analyze the effects of the RP on post-COVID-19 syndrome patients with respect to objective and subjective functional capacity, balance, and musculoskeletal strength. **Methods**: A prospective interventional trial was conducted before and after this phase. Patients were referred to the hospital with a confirmed diagnosis of SARS-CoV-2 and subsequently directed to the RP. These patients underwent an 8-week pulmonary rehabilitation program (45-min sessions 3 times/week). Each session consisted of stationary cycle-ergometer and resistance musculoskeletal exercises tailored to individuals’ performance. They were evaluated pre- and post-PRP using the maximal handgrip strength (HGS) test, timed up-and-go test, 6-min walk test and its derived variables, and Duke Activity Status Index questionnaire. **Results**: From 142 hospitalized patients admitted with a diagnosis of SARS-CoV-2 infection, 60 completed the program, with an attendance rate of 85%. Nineteen patients were categorized as severe/critical, with a significantly higher hospital stay, compared to mild/moderate patients, and there were no differences in terms of sex distribution, age, or BMI between groups. Compared to the pre-PRP evaluation, both groups showed significant (*p* < 0.001) improvements in TUG, HGS, DASI D6MWT, 6MWS, and DSP variables after the PRP conduction. In addition, the groups exhibited similar improvement patterns following PRP (intragroup analysis), with no intergroup differences. **Conclusions**: RPs promote both objective and subjective functional capacity in patients with post-COVID-19 syndrome, with no difference in improvement regardless of the severity of the initial infection.

## 1. Introduction

The last few years have been challenging for healthcare professionals, managers, scientists, and the general population [1]. COVID-19, caused by direct contact with SARS-CoV-2, has caused a significant burden on healthcare systems worldwide. The infection may manifest with symptoms such as fever, dyspnea, cough, mental confusion, proteinuria, and skeletal muscle wasting as part of the systemic response [2]. Severe (14%) and critical (5%) cases often develop severe pneumonia and respiratory failure, requiring supplemental oxygen or mechanical ventilation during hospitalization. In addition, these patients are more likely to experience long-term sequelae [3], further increasing the demand for health services.

Furthermore, the immediate acute convalescent period is marked by several symptoms [4], the most common being reduced functional capacity and minimal-effort dyspnea [5], which significantly impact daily functioning, as assessed using the international classification of functioning and functional capacity evaluations [6]. SARS-CoV-2 convalescence may present with multiple symptoms that persist for months and cannot be explained by other pathophysiological causes [7], a condition recognized as post-COVID-19 syndrome. Promoting measures that enable full restoration or rehabilitation of functional status is crucial to minimizing the social and financial impacts of the infection. Thus, complete restoration of impaired function is anticipated when patients undertake a rehabilitation program (RP).

The pulmonary RP, a well-established non-pharmaceutical measure for chronic respiratory disease patients, is a comprehensive intervention based on individual and extensive evaluation of patients. A key component of the RP is the implementation of physical exercises, typically lasting between 8–12 weeks, aiming at relieving symptoms, improving physical condition, enhancing functional capacity, and boosting quality of life [8]. 

A RP is reportedly an effective measure for post-COVID-19 syndrome patients [9]. It can improve exercise capacity, enhance lung function, and reduce muscular fatigue [10]. More specifically, the effects of a pulmonary rehabilitation program (PRP) have been observed in mild-to-moderate SARS-CoV-2 infection cases [11]. However, there is a paucity of studies that present data on these effects in moderate-to-severe cases [12]. It is crucial to analyze the improvements in both mild-to-moderate and severe-to-critical post-COVID-19 syndrome patients, as improvements of different magnitudes can take place between these groups; additionally, this might guide the implementation of physical exercises tailored to individuals’ performance and severity of initial SARS-CoV-2 infection. Moreover, it is important to analyze whether a minimum 8-week program is effective in improving functional capacity and muscular performance. Thus, the present study aimed to assess the efficacy of an 8-week physical exercise program based on PRP in post-COVID-19 syndrome patients and to compare dynamic balance, functional capacity, and muscle strength between those with mild/moderate initial infection and those with severe/critical SARS-CoV-2 initial infection.

## 2. Materials and Methods

### 2.1. Study Design 

We conducted a prospective interventional, before and after trial, involving participants referred to a RP between 1 and 2 months after being hospitalized for SARS-CoV-2 infection between May 2020 and December 2021 and discharged. Their symptoms were persistent with those of post-COVID-19 syndrome. This study was conducted in a hospital setting and approved by the ethics committee (#35437020.0.0000.5076). It has also been registered at Clinicaltrials.org under the identification COVID-19 PULMONARY REHAB NCT04982042. 

### 2.2. Data Collection and Participants

We included patients aged 18 to 75 years ikd who had medical indications and approval for immediate follow-up in a RP following a SARS-CoV-2 acute infection confirmed through a reverse transcription-polymerase chain reaction. These patients also exhibited significant major pulmonary convalescence (dyspnea and/or exertional breathing or fatigue on exertion). Individuals with other indications for RPs (not recently to recent SARS-CoV-2), those who were clinically unstable, or those with physical dependence and/or neurologic, orthopedic, or psychiatric conditions that could impede participation in the RP were excluded. To describe the effects of the severity of SARS-CoV-2 infection on functional outcomes, the patients were divided into two groups according to the severity of the initial infection: mild/moderate for those without oxygen supplementation or hospitalization and severe/critical for those hospitalized and under oxygen supplementation.

### 2.3. Functional and Physical Evaluation

All patients were initially evaluated between 1 and 2 months after hospital discharge. Patients were evaluated at both the beginning and the end of the rehabilitation program, and these assessments were conducted by an experienced physiotherapist. This assessment included collecting demographic data (weight, height, and age), information about the severity of SARS-CoV-2 infection (use of supplemental oxygen, noninvasive, or invasive mechanical ventilation), and coexistent comorbidities. Dynamic balance was measured with the timed up-and-go (TUG) test. For this measurement, the time (seconds) spent rising from a chair, walking 3 m, turning around, and sitting down in the same chair, as fast as possible without running, were recorded [13]. TUG times exceeding 12–15 s were considered a cut-off value indicating mobility issues or potential health risks [14].

The measurement of skeletal muscle performance included an evaluation of one maximal voluntary contraction (1-MVC) [15] and maximal handgrip strength (HGS). The 1-MVC, a dynamic test, is a useful tool for assessing the maximum weight an individual can lift during a voluntary contraction without compensatory movements. It was specifically used to quantify the initial resistance set for the muscular strengthening regime proposed by the RP. We evaluated elbow flexion and knee extension, progressively increasing the weight until participants could no longer perform the contraction without compensatory movement, with 1 min of rest between each increment. The initial resistance for muscular strengthening was set at 50% of the initial 1-MVC. 

To measure HGS, a palm-handgrip dynamometer (*JAMAR*, hydraulic dynamometer, Saehan) was used. It measures the highest voluntary strength of the hand and may serve as a risk-stratifying screening tool for muscular strength and neuromuscular functioning. [16] The patients were asked to remain seated in a chair with their feet completely flat on the ground, resting their elbows on the chair’s arms at 90° flexion, and instructed to perform maximal HGS with both arms. Patients were allowed three attempts for each arm, with a 1-min interval between attempts, and the highest value for the dominant arm was recorded (DHGS).

Functional capacity was measured using the Duke Activity Index Scale (DASI) questionnaire and the 6-min walk test (6MWT). The DASI questionnaire, as described elsewhere [17], is used to assess self-related quality of life a week before its application. It is a 12-item scale where the patients are questioned about their ability to perform personal care, ambulation, household tasks, sexual function, and recreational activities. For each item evaluated, a weight is assigned for each answer, which can vary from 1.75 to 8.0, reflecting the task intensity. Thus, the final self-reported functional capacity may vary from very low (scores below 16) to low (scores from 16–30), moderate (31–45), and high functional capacity (58.2 points). 

The 6MWT, a submaximal exercise test, was conducted following published guidelines [18] and consisted of the evaluation of the distance walked for 6 min, without running, performed in a 30 m corridor. We recorded the distance walked during the 6MWT (D6MWT) and 6MWT-derived variables. 6MWT speed (6MWS) was calculated by dividing the distance by the total walking time (360 s) [19]. The distance-saturation product (DSP) was calculated as the product of the final distance walked and post-exercise SpO_2_ [20]. The heart rate and peripheral oxyhemoglobin saturation were measured immediately before and after testing. For patients presenting with desaturation during the 6MWT and/or RP sessions, supplemental oxygen was offered via a nasal cannula until SpO_2_ > 92%. 

### 2.4. Pulmonary Rehabilitation Program

Physical exercises for the RP were conducted by an experienced PT who did not have access to the initial evaluations. The conduction of physical exercises program was based on international guidelines proposed for chronic obstructive pulmonary disease (COPD) patients, as described elsewhere [8]. Briefly, the patients were enrolled in an 8-week program three times per week in 45-min sessions. During the RP period, a maximum absence of 15% was allowed (four sessions), provided that no more than two consecutive sessions were missed. Each session consisted of a 15-min warm-up and cool-down on a stationary bicycle, interspersed with progressive resistance exercises for muscle strengthening in the following movements: elbow extension/flexion, shoulder abduction/flexion, knee extension/flexion, hip extension/flexion, and abduction/adduction. The resistance (measured in Watts) on the stationary bicycle during warm-up was set to maintain the heart rate at 40 to 60% of the maximal predicted value [21], which was gradually increased up to 70% as tolerated by the patients. In the cool-down component, the patients were allowed to perform without resistance. For resistance exercises, the patients were asked to perform 30 repetitions, divided into three sets of 10 repetitions of each movement. The initial resistance was determined as 50% of MVC measured, and the resistance was increased weekly, from 0.5 kg, until each patient reached their tolerance level and/or began using compensatory movements during the exercise. Sessions were performed with 1 min of rest between exercises. Based on the overload principle for PRP [8], we proposed an initial lower level of resistance with progressive improvements over the weeks. Thus, low-intensity exercises were initially prioritized to gradually prepare the muscles over the weeks for higher intensities. 

### 2.5. Statistical Analysis

The Strengthening the Reporting of Observational Studies in Epidemiology checklist was used to improve the data presentation quality [22]. The final sample size (n) was estimated by considering the population of SARS-CoV-2 patients referred to the PRP from our hospital setting immediately after being hospitalized for COVID-19. To calculate the sample size, we used a 95% confidence interval and a 0.05 margin of error.

Numerical variables are described as mean ± standard deviation (mean ± SD). The Kolmogorov–Smirnov test was used to assess data normality. For intragroup comparisons, paired *t*-tests were employed for normally distributed data, and the Wilcoxon signed-rank test was utilized for non-normally distributed data. For intergroup comparisons, independent *t*-tests were used for normally distributed data, and the Mann–Whitney U test was applied for non-normally distributed data.

The data were analyzed using IBM SPSS Statistics for Windows, Version 20.0 (IBM Corp. Released 2011. Armonk, NY, USA). Statistical significance was set at *p* < 0.05.

## 3. Results

Initially, 142 patients with a diagnosis of SARS-CoV-2 infection were referred for hospital stay. Of these, 72 patients with persistent post-COVID-19 symptoms and major pulmonary convalescence were referred for an initial evaluation for the RP. Twelve patients did not complete the program, eight abandoned it for economic reasons, and four were clinically unstable at the initial evaluation (two patients presented with a SpO_2_ at rest <78%, which was not corrected even after the introduction of supplemental oxygen via a nasal cannula (SpO_2_ < 92%), and two others displayed palpitations). Sixty patients completed an 8-week RP. All patients completed the RP, with an absence rate of <15%. A slightly higher number of males than females were included in the final sample. Most were in their 50 s and overweight, with an average hospital stay of approximately 2 weeks. Table 1 presents the detailed data of the 60 patients who completed the program. 

In the severe/critical group, 17 patients were placed under noninvasive ventilation, and 12 underwent invasive mechanical ventilation during their hospital stay. Only one patient (initial mild/moderate COVID-19 infection, previously obese, with uncontrolled blood pressure, diabetes mellitus, and dyslipidemia) presented with exercise-induced desaturation at the initial evaluation, which was corrected with oxygen supplementation via nasal cannula. Another patient (initial several/critical COVID-19 infection, previously obese, with a history of chronic lung disease) who presented with exacerbation of their condition a few days after the end of the RP exhibited exercise-induced desaturation only at the final RP evaluation; this patient was further followed at the pneumology unit ward for a full investigation. 

Table 2 outlines the intra-and intergroup comparisons of mild/moderate initial COVID-19 versus severe/critical cases. Intragroup analysis indicated significant improvements in all assessed variables in both subgroups. However, no significant differences were found between the groups for any of the measured variables. 

Although not statistically significant, the delta gains, i.e., the absolute differences in TUG, DASI score, and all 6MWT variables measured between post- and pre-RP—were higher in the severe/critical group. Regarding functional status as measured by the 6MWT, 11/19 (58%) patients in the severe/critical group had pre-RP D6MWT values below the predicted performance (<80%), with all normalizing their performances in the post-RP evaluation. In the mild/moderate group, 13/41 (32%) patients had pre-RP D6MWT values below the predicted values, and all but one patient, who ultimately reached 78% of the predicted value, normalized their performances post-RP.

## 4. Discussion

Our study included patients in the post-acute stage of COVID-19 infection who underwent an 8-week RP and experienced improvements in functional mobility/dynamic balance, HGS, and functional status (6MWT, its derived variables, and DASI). Furthermore, our detailed subgroup analysis revealed that both the mild/moderate and severe/critical groups showed improvements in the evaluated parameters after the RP. However, no significant differences were detected between groups.

In the analysis of demographic variables and length of hospital stay between the groups, only the latter was significantly higher in the severe/critical group. Longer hospital stays, mainly in the *ICU* ward, increase the susceptibility to muscle deconditioning and weakness, culminating in major impacts on muscle function and functional capacity. Thus, the severe/critical group, presenting with a significantly longer hospital stay, was more exposed to factors that have important implications for functional capacity [23], particularly longer bed rest. Additionally, SARS-CoV-2 infection may lead to a reduction in this capacity, causing further deterioration of patients’ functional abilities [22]. 

Regarding the studied functional variables, the TUG test showed a significant reduction in time after the RP, indicating improvements in functional mobility and dynamic balance in COVID-19 patients [24]. The mild/moderate subgroup did not have pre-RP values indicating mobility issues or potential health risks, as their times were below 8.4 s. Conversely, the severe/critical subgroup had pre-RP times exceeding this value [25]. However, comparisons of pre- and post-RP values showed significant improvements for both groups. Corroborating our findings, previous studies have also demonstrated the efficacy of the RP on TUG performance [26]. We believe the improved performance post-RP is attributable to enhancements in skeletal muscle strength, aerobic capacity, and motor control.

Our HGS results were similar to other pertinent studies with respect to COVID-19 patients [23]. As presented in Table 2, only the mild/moderate subgroup showed a significant improvement in handgrip force after the RP. A more comprehensive analysis of both groups showed that 5/41 (12%) patients in the mild/moderate group had handgrip force lower than 80% of the predicted value [16] at the pre-RP evaluation. At the post-RP evaluation, all patients achieved typical predicted values. Conversely, 3/19 (16%) in the severe/critical group had HGS less than 80% of the predicted value at the initial evaluation, but all normalized their values after the RP. 

Both functional measurements (DASI and 6MWT) improved after the RP. The self-perception of functional status, established through the DASI score, showed that patients noticed an impairment in their functional status at the initiation of the RP. The higher mean values found following the RP suggest that this questionnaire may have reached a ceiling effect (maximum score: 58.2). Thus, we hypothesized that the questionnaire was not sufficiently comprehensive to be able to accurately measure the reported gain in functional status. Consequently, 16/19 (84%) patients from the mild/moderate group and 38/41 (93%) from the severe/critical group exhibited maximal DASI scores at the post-RP evaluation.

The mean distance covered in the 6MWT improved significantly after the RP. One study underscored a clinically significant improvement of 30 m in the 6MWT for COPD patients [27]. To emphasize the significance of this clinical improvement, the mean distance covered by our patients at the initial evaluation was below the predicted mean value of approximately 82.8 m. After the RP, the mean distance covered exceeded the predicted mean value by approximately 32.3 m. The mild/moderate subgroup patients had an average predicted value of 87% at initial evaluation, and they improved by up to 104% [24] in the post-RP evaluation (improvement of 94.5 m). The severe/critical subgroup patients had an average predicted value of 83% at initial evaluation and reached 106% of the predicted value [28] (an improvement of 129.8 m).

Furthermore, the 6MWT-derived variables (6MWS and DSP) significantly improved after PRP. A comparison of our results with similar studies on chronic lung disease patients indicated that our patients performed a higher 6MWS [29], higher DSP, longer D6MWT, and higher post-exercise SpO_2_ levels [29,30]. As improvement was observed in these variables after the RP, the functional status impairment of COVID-19 patients is transitory and less critical than that in chronic lung disease patients, for whom respiratory, musculoskeletal, and functional impairments are persistent or permanent. 

Lower DSP values, as observed in chronic lung diseases, reflect impairments in pulmonary exchange or skeletal muscle metabolism. We found significant improvements in this measure in both subgroups and attributed these gains to improvements in the distance walked in 6MWT. The patients did not experience desaturation during the 6MWT and maintained a post-6MWT mean SpO_2_ of >96%, corroborating this conclusion. The DSP delta gain (value post-PRP minus value pre-PRP) was greater in the severe/critical subgroup. 

A similar study (with a small sample size) investigating the efficacy of pulmonary rehabilitation in both mild/moderate and severe/critical COVID-19 patients presented similar results but analyzed the effects through an inpatient RP [12]. By conducting a similar comparison with a larger sample size, in accordance with the prevalence of cases by disease severity [3], and considering an outpatient RP, our study demonstrated the efficacy of the RP even when conducted over a shorter period (8wk). From the international standpoint statements for rehabilitation in COPD patients, the program may last from 8 to 12 weeks [8]. Although our study conducted the RP for a shorter duration, significant improvements in measured variables were observed for all patients. We preferred to perform the RP in a shorter period to allow for a higher turnover rate among patients (especially during periods of increased COVID-19 cases). This approach also addressed economic considerations and reduced transportation expenses for patients. Additionally, several studies affirm the importance of the RP for patients with long COVID, as it helps mitigate long-term [12,31] fatigue, dyspnea, and functional impairments, thereby improving overall quality of life.

Intergroup analysis revealed no significant differences in the variables studied. Nevertheless, the delta gain, that is, the absolute difference between post- and pre-RP values, was higher in the severe/critical subgroup for TUG, DASI score, and all 6MWT variables; hence, this group may have experienced more significant losses in musculoskeletal and overall capacity due to COVID-19 than the mild/moderate subgroup. Thus, DASI score and 6MWT variables showed lower pre-RP values, with higher values observed post-RP in this group, except for the DSP, which did not follow this trend. It is important to note that both group patients were of similar age; therefore, age did not influence the performance of 6MWT. In our study, 24/60 patients were mechanically ventilated during an acute COVID-19 infection, and 23 were overweight/obese. 

### Study Limitations

The primary limitation of this study was the absence of a control group. COVID-19 may lead to the transitory impairment of overall pulmonary, cardiac, and musculoskeletal functions. Therefore, we did not compare a probable transitory damage pattern with the chronic impairments observed in other conditions that are also addressed by the RP. Additionally, a cardiopulmonary exercise test should be administered to better characterize the damage to the patients’ maximal exercise capacity. This analysis would better describe the eventual initial ventilatory/cardiovascular or deconditioning limitations at the start of the RP and occasionally at the end, especially in severe/critical patients with post-acute COVID-19 [32]. Furthermore, using the SF-36 [33] instead of the DASI would be more appropriate because the post-RP DASI analysis might not have adequately evaluated the improvement owing to a ceiling effect. However, as the DASI measures the quality of life in the period before its application, we initially found it to be more appropriate. In addition, the evaluation of pulmonary function with stratification of impairment would add relevant information about pulmonary function and its progression post-RP. Finally, a higher number of patients, especially for the severe/critical group, should be included to obtain more balanced samples between the groups. 

Determining the dropout rate from the initial screening to RP implementation is important. This finding was substantial and may have influenced the final statistical analysis. Moreover, in COVID-19’s natural course, some patients may experience spontaneous remission. However, this change may have been impossible to accurately identify owing to clinical presentation and progression heterogeneity. Ultimately, we found that our final sample accurately represented the identified RP effects, assuming an over 60% prevalence of post-COVID-19 symptoms in infected patients. Among hospitalized patients, 85% of patients may experience these symptoms. Thus, our sample may have had a longer convalescence post-SARS-CoV-2 infection, and the deleterious effects were counterbalanced by the RP. 

## 5. Conclusions

A RP is a useful intervention that facilitates the restoration of handgrip measurements and functional status in post-acute COVID-19 patients. Mild/moderate and severe/critical patients experienced similar gains after undertaking the RP.

## Figures and Tables

**Table 1:** Total and subgroup demographic data of post-acute COVID-19 patients who completed a PRP (*n* = 60). Values expressed by mean ± standard deviation.

	Mild/Moderate *n* = 41	Severe/Critical *n* = 19	Total *n* = 60	*p*-Value
Hospital stay (days)	11.4 ± 11.1	25.2 ± 18.3	15.8 ± 15.1	0.005
Sex (Male/female)	20/21	12/7	32/28	NS
Age (years)	52.9 ± 12.9	52.5 ± 11.3	54.4 ± 12.2	NS
BMI (kg/m^2^)	27.2 ± 5.8	27.3 ± 3.3	27.2 ± 5.1	NS

BMI: body mass index; PRP: pulmonary rehabilitation program; NS: not significant.

**Table 2:** Pre- and post-pulmonary rehabilitation program (PRP) evaluations from all assessed patients and subgroup presentation: mild/moderate and severe/critical SARS-CoV-2 infection. Values expressed by mean ± standard deviation.

		Mild/Moderate *n* = 41	Severe/Critical *n* = 19	Total *n* = 60
TUG (seconds)	Pre-RP	8.2 ± 1.9	8.5 ± 1.7	8.2 ± 1.8
	Post-PRP	6.3 ± 1.2	6.0 ± 0.9	6.2 ± 1.1
Intra-group *p*-value		<0.001	<0.001	<0.001
DHGS (kgf) (% predicted) DHGS (kgf) (% predicted)	Pre-PRP	30.4 ± 11.5	32.0 ± 11.6	30.9 ± 11.5
		(114 ± 28%)	(119 ± 35%)	
	Post-PRP	35.3 + 9.7 (134 ± 22%)	36.0 ± 8.7 (131 ± 32%)	35.4 ± 9.3
Intra-group *p*-value		0.04	ns	0.02
DASI (score)	Pre-PRP	30.6 ± 14.5	27.5 ± 15.0	29.5 ± 15.3
	Post-PRP	56.5 ± 3.0	56.4 ± 3.0	56.3 ± 5.9
Intra-group *p*-value		<0.001	<0.001	<0.001
D6MWT(meters)	Predicted (meters)	561.7 ± 48.5	564.1 ± 45.5	563.4 ± 47.2
	PRP (meters) (% predicted)	489.0 ± 87.4 (89 ± 16%)	466.8 ± 100.9 (83 ± 19%)	482.0 + 91.7
	PRP (meters) (% predicted)	583.5 ± 84.7 (106 ± 16%)	595.6 ± 75.1 (105 ± 15%)	587.3 ± 81.3
Intra-group *p*-value		<0.001	<0.001	<0.001
6MWS (meters/seconds)	Pre-PRP	1.4 + 0.3	1.3 + 0.3	1.3 + 0.3
	Post-PRP	1.6 + 0.2	1.7 + 0.2	1.6 + 0.2
Intra-group *p*-value		<0.001	<0.001	<0.001
DSP (m%)	Pre-PRP	474.0 + 83.5	444.0 + 104.3	464.5 ± 90.8
	Post-PRP	565.7 + 83.8	565.5 + 88.2	568.7 ± 81.5
Intra-group *p*-value		<0.001	<0.001	<0.001

BMI: body mass index; TUG: timed up-and-go; DASI: Duke Activity Status Index; DHGS: dominant handgrip strength; D6MWT: distance walked on the 6-min walk test; 6MWS: speed during the 6MWT; DSP: distance-saturation product.

## Data Availability

Patient data were anonymous, and owing to the ethical requirements and hospital policies where the data were collected, the corresponding author was the only one to possess full data access.
